# A 98-Year-Old Male With Paroxysmal Atrial Fibrillation Treated for COVID-19 at Home

**DOI:** 10.7759/cureus.30653

**Published:** 2022-10-25

**Authors:** Fabrizia Farolfi, Stefania Cavazza, Andrea Mangiagalli, Luigi Cavanna

**Affiliations:** 1 General Practice, Azienda Unità Sanitaria Locale della Romagna, Solarolo, ITA; 2 Internal Medicine, Arianna Anticoagulazione Foundation, Bologna, ITA; 3 General Practice, Agenzia di Tutela della Salute Città Metropolitana di Milano, Milano, ITA; 4 Onco-hematology, Hospital of Piacenza, Piacenza, ITA

**Keywords:** dietary supplements, anti-inflammatory drugs, early treatment response, old patients, covid-19 retro

## Abstract

In the absence of evidenced-based guidelines for early home treatment of COVID-19, some Italian groups of volunteer physicians (both general practitioners (GPs) and hospital doctors) virtually gathered themselves to discuss the best available evidence and develop shared schemes of therapy. We present the case of a 98-year-old unvaccinated male on chronic anticoagulant therapy with dabigatran for paroxysmal atrial fibrillation (AF), who has been successfully treated for COVID-19 at home, according to one of the multidrug treatments proposed, since hospital admission was not feasible. At the very beginning of symptoms, anti-inflammatory drugs, vitamin D, and adjuvant dietary supplements (quercetin, vitamin C, zinc, and vitamin K2) were administered, followed by dexamethasone and antibiotic therapy, according to the evolving clinical conditions. Gastroprotection with omeprazole was added. Eventually, our patient fully recovered, thus suggesting that careful home assistance under strict medical supervision can be successful, even in a very old subject with comorbidities, particularly if early treatment simultaneously addressing inflammation, hypercoagulation, and viral replication is started.

## Introduction

During the last two years, with the outbreak of the COVID-19 pandemic, physicians had to face the lack of fully approved evidence-based treatments for this new disease. The scarcely available randomized controlled trials (RCTs) were mainly performed in hospital settings and only involved patients in an advanced stage of the disease. The lack of evidence regarding the early phase of COVID-19 home treatment leads the Italian Ministry of Health to issue guidelines recommending a “wait and see approach” while suggesting only symptomatic treatment for fever and pain [1]. Moreover, in Italy, monoclonal antibodies and antivirals are approved only for high-risk patients within five days from the onset of symptoms [2], but they may well be hardly available and have limitations of use due to side effects and interactions with other drugs often used in elderly people with comorbidities. Besides, the oral antiviral nirmatrelvir/ritonavir (Paxlovid®) had not been licensed in Italy yet at the time we treated our patient at the beginning of January 2022. Meanwhile, many general practitioners (GPs) considered it unethical to neglect any intervention based on safe therapies following relevant pathophysiological rationales, especially in very old people [3]. Observational studies showed that early home treatment with nonsteroidal anti-inflammatory drugs (NSAIDs), possibly associated with vitamins and micronutrients, is effective in reducing hospitalization and symptom duration [3-5], and that’s why some multidrug approaches were proposed [3,4,6,7]. At the very beginning of the worldwide pandemic outbreak, in Italy, healthcare professionals had to face such a new and aggressive disease that some of them created voluntary groups of doctors aiming to discuss the possible best scientific evidence for a helpful therapy, thus developing shared and efficient schemes of early therapy. Consequently, they gained a vast and thorough experience in treating thousands of home patients through phone calls, video calls, and home visits as well. One of the largest was “Early Home Therapy for COVID-19” group (https://www.terapiadomiciliarecovid19.org) [8].

Here, we present the case of a 98-year-old unvaccinated male with paroxysmal atrial fibrillation (AF), who was successfully treated at home in early January 2022 by his GP. At that time, the new winter delta wave peak of contagions forced hundreds of patients to pack into hospitals, so physicians had to find a way to organize home hospital care.

## Case presentation

A 98-year-old Caucasian male sought advice from his GP for fever and cough on January 6, four days before he had been in contact with a SARS-CoV-2-positive person with a probable delta virus variant. His medical history included hypertension, mild renal impairment, prostatic hypertrophy, biliary sludge, and paroxysmal atrial fibrillation on long-term anticoagulation therapy with dabigatran. Recent blood tests revealed a normal blood count, electrolytes, uric acid, glycemia, and hepatic function and a mildly decreased renal function (creatinine: 1.34 mg/dl; glomerular filtration rate: 44 ml/minute). On the same day of the first contact with his GP, he was discovered to be positive using an antigenic SARS-CoV-2 home test (subsequently confirmed by reverse transcription polymerase chain reaction) and was immediately started on anti-inflammatory drugs (acetylsalicylic acid {ASA}: 330 mg twice per day {bid}),vitamin supplements (vitamin C: 1 g three times a day, vitamin D: 10,000 IU once a day {od}, vitamin K2: 100 mg bid), quercetin: 250 mg bid, and zinc: 25 mg bid, in addition to his usual therapy (dabigatran: 110 mg bid; furosemide: 12.5 mg od; lercanidipine: 10 mg od; ursodeoxycholic acid: 150 mg bid; dutasteride: 0.5 mg od; tamsulosin: 0.4 mg od). Afterward, the patient was tele-monitored several times a day by his GP.

**Figure 1:**
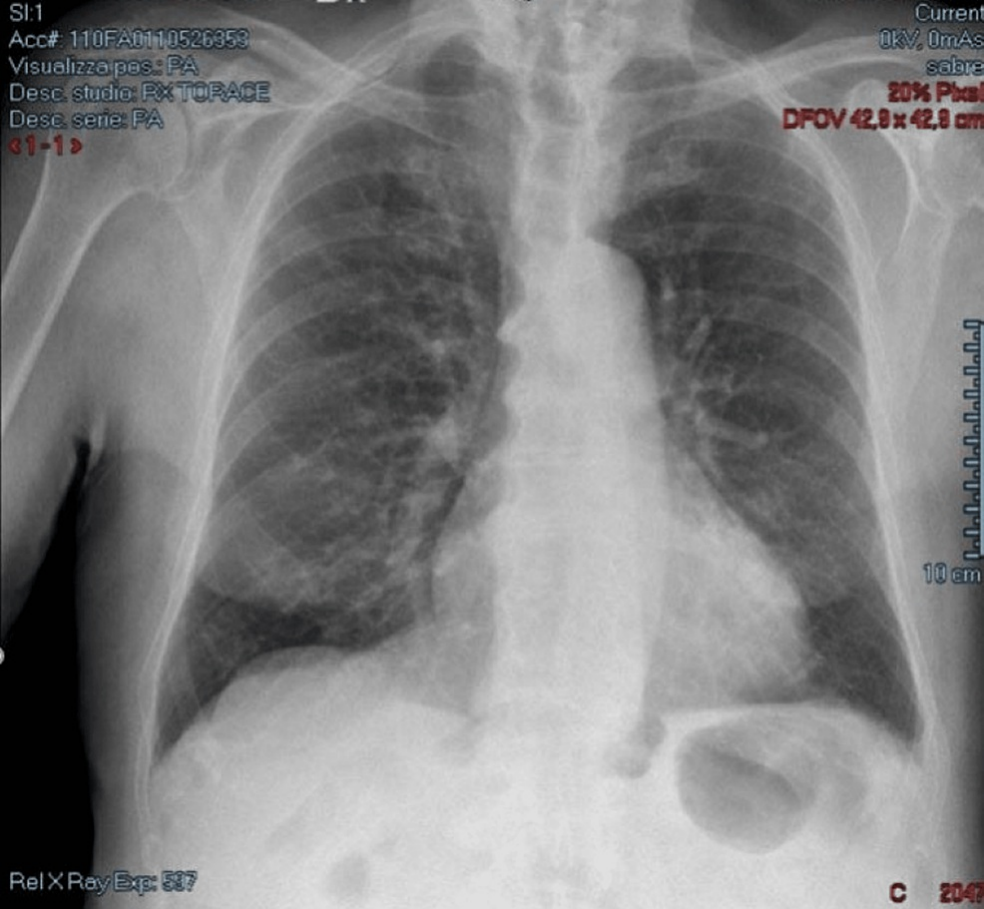
Chest X-ray showing interstitial pneumonia

From day 1 to day 3, his symptoms were mild cough and fever. On day 4, body temperature raised up to 39°C along with dyspnea and mental confusion. During the day, his blood oxygen saturation (SpO_2_) in room air rapidly decreased from 98% to 88%, so oxygen supplementation through the nasal cannula was promptly started at home (4 l/minute). At the same time, corticosteroid therapy with dexamethasone 4 mg intramuscularly (im) bid was administered along with gastroprotection (omeprazole: 20 mg bid). On day 5, an antibiotic was added (ceftriaxone: 1 g im od) for suspected bacterial superinfection, and acetylsalicylic acid was stopped because of a self-limiting episode of hemoptoe. From day 5, dyspnea, confusion, and fever steadily decreased, and on day 12, oxygen supplementation was stopped. Precisely, oxygen was administered continuously at low flux (4 l/minute) for a week and was stopped only when room air saturation was steadily kept over 96%. The patient gradually improved, and every treatment was stopped on day 14. On day 20, an antigenic SARS-CoV-2 test was performed, which was negative. During the COVID-19-positive phase, no blood or radiological test was done. A chest X-ray was carried out soon after clinical healing, which showed diffused lung interstitial thickening (Figure 1). After one month of follow-up, GP visit revealed that the patient had clinically recovered and following routinely performed blood tests (February 28) did not reveal any worsening; in particular, renal function was preserved (creatinine: 1.03 mg/dl; glomerular filtration rate: 60 ml/minute), and blood cell count was normal (hemoglobin: 13.3 g/dl). Since the patient was completely asymptomatic, chest X-ray was not reassessed. At the time of writing, in September 2022, the patient is alive and enjoys good health. Three months after healing, the patient was given adequate counseling to get vaccination against COVID-19.

## Discussion

COVID-19 is a multisystemic disease that involves hyperinflammation and hypercoagulation, which are closely connected to each other. Based on these pathophysiological mechanisms of the disease, various multidrug approaches were proposed for early outpatient treatment of mild COVID-19 [3,4,6,7]. Nonsteroidal anti-inflammatory drugs (NSAIDs) administered at the beginning of the symptoms have shown to significantly reduce hospitalization and disease duration in observational studies [3-5,9] and in a small open-label RCT [10]. While waiting for more solid evidence and generally approved guidelines, many GPs decided to treat outpatients at the first stage of the disease with commonly available over-the-counter drugs that had been previously suggested to prevent the progression of the disease.

In the case described, an unvaccinated 98-year-old male with multiple age-related comorbidities was treated at home for COVID-19, since hospitalization was not feasible. Symptoms were mild at presentation. Due to local regulation and organization, there were difficulties to obtain access to antiviral drugs or monoclonal antibodies, which were only available as hospital-based intravenous therapies. He was on chronic treatment with dabigatran, a direct oral anticoagulant (DOAC), for paroxysmal AF, which was continued according to Italian Medicines Agency (AIFA) recommendations [2]. At the beginning of January 2022, home treatment with oral antivirals (Paxlovid®) had not been licensed in Italy yet, but in any case, it would have been contraindicated in our patient given the concomitant therapy with dabigatran. Since macrothrombosis and microthrombosis are implicated in adverse outcomes after SARS-CoV-2 infection, the role of anticoagulants in COVID-19 patients has been extensively investigated; hence, recommendations were issued, but they are mainly related to low-molecular-weight heparin (LMWH) in hospital settings [11]. Studies on the role of ongoing chronic oral anticoagulation in reducing mortality in COVID-19 patients gave conflicting results. DOACs have been postulated to be less protective than LMWH due to the missing anti-inflammatory effect, mainly considering the constant inflammatory mechanism at the base of thrombosis and the progression of the disease [11]. To provide an anti-inflammatory action, acetylsalicylic acid (ASA) was administered at a dose of 330 mg bid, which was suggested to reduce plasma levels of inflammatory cytokines [4,6,12]. Moreover, in this patient, affected by diffuse though moderate arteriosclerotic disease, the antiplatelet effect of ASA was expected to further contrast the risk of thrombosis enhanced by COVID-19. The bleeding risk was increased by both the concomitant use of oral anticoagulant and the old age of the patient, but with appropriate gastroprotection, no major bleeding was observed even if a mild and self-limiting hemoptoe episode occurred that lead to the discontinuation of ASA.

In addition to these, our patient received vitamin D3 supplementation at high dosage (10,000 IU od), which was proven to favor an anti-inflammatory environment and improve a cytotoxic response against the virus [13]. In the last two years, many studies highlighted the potential role of vitamin D supplementation in enhancing the immune response against SARS-CoV-2. A meta-analysis showed that vitamin D supplementation improves the outcome of hospitalized patients with COVID-19 [14]. A single small RCT including outpatients with mild to moderate COVID-19 found that the intervention arm (vitamin D3 10,000 IU/day for 14 days) showed significantly fewer symptoms on the seventh and 14th day of follow-up compared to controls [15]. Many trials are still ongoing (www.clinicaltrials.gov), the vast majority of which compare high vitamin D doses with low doses (but not versus placebo), since it is considered unethical “to prevent patients from a potentially effective treatment” [13]. Vitamin K2 was added to enhance vitamin D function and to contrast potential adverse effects such as hypercalcemia.

As adjuvant to anti-inflammatory and immune-regulating drugs, quercetin, a flavonoid with antiviral properties, was added together with vitamin C and zinc for their synergistic effects [16]. These micronutrients with antioxidant potential are expected to contrast the oxidative stress, which appears to be a main cause of cell damage and disease progression [16]. Two small prospective RCTs showed a reduction in hospital admissions and in number of deaths in patients who received quercetin supplementation at an early stage of COVID-19 [17,18]. Based on previous evidence, several clinical trials investigated high-dose intravenous vitamin C along with other treatments in hospitalized patients with severe COVID-19 but ended with conflicting results. Very few data are available on outpatients with mild to moderate COVID-19 even if a reanalysis of COVID A-Z trial showed that oral vitamin C plus zinc supplementation may increase the recovery rate of SARS-CoV-2-infected patients [19]. Many trials on vitamin C in COVID-19 patients are still ongoing (www.clinicaltrials.gov), but testing of multidrug approaches is lacking. Notably, a still-recruiting Canadian RCT is investigating the same combinations of micronutrients as reported here (vitamin D, vitamin C, zinc, and vitamin K2), at very similar dosages in early COVID-19 outpatients [20].

On day 4, due to the worsening of respiratory symptoms and the drop of SpO_2_ to 88%, hospitalization was highly considered as a valid option, but the rapid improvement of the clinical conditions with O_2_ supplementation (which was delivered in advance and already available at bedside) and dexamethasone im suggested that it was safer for the patient to be treated at home instead of trying to reach a hospital. In those days, hospitals were overwhelmed with COVID-19 patients, and a long wait was expected before being admitted to the emergency room without certainty of a bed in a hospital ward. Oxygen supplementation with nasal cannula was given at low flux (4 l/minute) according to the patient’s need based on SpO_2_ monitoring; after one week, it was stopped since room air oxygen saturation was kept steadily over 96%. Clinical conditions rapidly improved, and two weeks after the onset of symptoms, the patient clinically recovered.

## Conclusions

Our case suggests that watchful home care under strict medical supervision can be successful even in a very old patient with comorbidities, particularly if early treatment simultaneously addressing inflammation, hypercoagulation, and viral replication is started. In this present case, the rapidly worsening clinical situation of an old and fragile patient forced his GP into quickly starting a strong treatment at home with close tele-monitoring that eventually led to a favorable outcome. The treatment scheme had been approved by a broad group of Italian physicians named “Early Home Therapy for COVID-19” (www.terapiadomiciliarecovid19.org) based on available evidence. Nonetheless, large clinical trials comparing different multidrug approaches for early home treatment of mild COVID-19 are missing though urgently needed in order to obtain widely approved therapies, which can prevent the unfavorable and rapid progression of the disease.
